# Reversed halo sign on chest computed tomography: a retrospective analysis of 286 cases

**DOI:** 10.1590/0100-3984.2025.0014-en

**Published:** 2025-07-17

**Authors:** Camila Soares Franco, Paula Terra Martins Almeida Amaral, Eduardo Kaiser Ururahy Nunes Fonseca, Tassia Regina Yamanari, Paulo Esrom Moreira Catarina, Alessandra de Pinho Pimenta Borges, Luiz Augusto de Moraes Pinheiro Filho, Felipe Freitas Camara, Márcio Valente Yamada Sawamura

**Affiliations:** 1 Hospital das Clínicas da Faculdade de Medicina da Universidade de São Paulo (HC-FMUSP), São Paulo, SP, Brazil; 2 Hospital Sírio-Libanês, São Paulo, SP, Brazil; 3 Hospital Israelita Albert Einstein, São Paulo, SP, Brazil

**Keywords:** Tomography, X-ray computed/methods, Respiratory tract infections/diagnostic imaging, Pulmonary embolism/diagnostic imaging, Lung diseases, interstitial/diagnostic imaging, Tomografia computadorizada/métodos, Infecções respiratórias/diagnóstico por imagem, Embolia pulmonar/diagnóstico por imagem, Doenças pulmonares intersticiais/diagnóstico por imagem.

## Abstract

**Objective:**

To characterize the main causes of the reversed halo sign (RHS) on computed tomography (CT) of the chest and its imaging features.

**Materials and Methods:**

This was a retrospective study reviewing all chest CT scans for which the report contained the term “reversed halo sign” among those performed between 2015 and 2020 at a tertiary care hospital.

**Results:**

A total of 286 cases were identified, and the corresponding CT images and clinical data were reviewed. In this population, the most common cause of an RHS was pulmonary infarction (in 42%), followed by cryptogenic organizing pneumonia (in 17%) and bacterial pneumonia (in 16%). In addition, the CT characteristics of the RHS were identified in various conditions, such as pulmonary thromboembolism with pulmonary infarction, in which the RHS was typically smooth-walled and solitary with a peripheral distribution.

**Conclusion:**

The RHS can be observed in many contexts, and its CT characteristics, in combination with the clinical picture, can help narrow the differential diagnosis.

## Abstract (pt)

**Objetivo:**

Caracterizar as principais causas do sinal do halo invertido (SHI) na tomografia computadorizada de tórax e suas características tomográficas.

**Materiais e Métodos:**

Foram revisadas, retrospectivamente, todas as tomografias computadorizadas de tórax realizadas em um hospital terciário, entre 2015 e 2020, que continham o termo “sinal do halo invertido” no relatório.

**Resultados:**

Foram encontrados 286 casos cujas imagens tomográficas e dados clínicos foram revisados. A causa mais comum na população estudada foi o infarto pulmonar (42%), seguido por pneumonia em organização criptogênica (17%) e pneumonia bacteriana (16%). Além disso, foram identificadas as características tomográficas do SHI, como no tromboembolismo pulmonar com infarto pulmonar, em que o SHI mais observado foi o de parede lisa, único e com distribuição periférica.

**Conclusão:**

O SHI pode ser observado em diversas condições, e suas características tomográficas aliadas ao contexto clínico do paciente podem ajudar a estreitar o diagnóstico diferencial.

## INTRODUCTION

On computed tomography (CT) of the chest, the reversed halo sign (RHS) is characterized by a round or oval area of ground-glass opacity completely or partially surrounded by a ring of consolidation in the lung parenchyma^(1)^. Although the RHS was first described in 1996 as a specific sign of organizing pneumonia^(2)^, subsequent studies demonstrated the presence of this sign in other conditions, such as pulmonary infarction, granulomatous infections, and sarcoidosis, thus making it a less specific finding^(3)^. The presence of smooth borders, nodular borders, or thickened walls in an RHS have been described as ancillary features in the differential diagnosis^(4^-^6)^.

During the coronavirus disease 2019 (COVID-19) pandemic, the RHS began to be observed in patients with the disease, mainly after the 10th day of infection with severe acute respiratory syndrome coronavirus 2 (SARS-CoV-2), possibly reflecting an evolution to the organizing pneumonia phase. Therefore, the RHS came to be considered a typical CT finding for COVID-19 in a clinical context consistent with the disease^(7)^. The diagnostic challenge of this finding was associated with the overlapping cases of pulmonary infarction, in addition to other causes, mainly in Brazil, where there is a significant prevalence of infectious granulomatous diseases.

The objective of this study was to perform a retrospective analysis of the RHS at our institution and compare the results with those in the literature, aiming to describe the diseases most related to this finding, the forms of presentation of an RHS, and its most prevalent characteristics in different diseases.

## MATERIALS AND METHODS

A search for the term “reversed halo” was conducted among the radiology reports of all consecutive chest CT scans acquired at our institution between 2015 and 2020. After this selection, the following clinical data were evaluated for each patient: age, sex, comorbidities, and the presence of COVID-19 at the time of the examination (in examinations performed in 2020). The definitive diagnosis of an RHS was defined based on the evaluation of the medical records, considering the clinical, biochemical, and pathology findings when available and, in some cases, ancillary studies such as CT angiography of the chest to investigate pulmonary thromboembolism, when that hypothesis was raised.

The CT images were evaluated in a random, blinded manner by two radiologists specializing in thoracic imaging, with one and three years of experience, respectively, working independently. The following imaging characteristics were described for each case: number, location, and distribution of the lesions; type of borders; and presence of nodules within the lesion.

**Figure 1:**
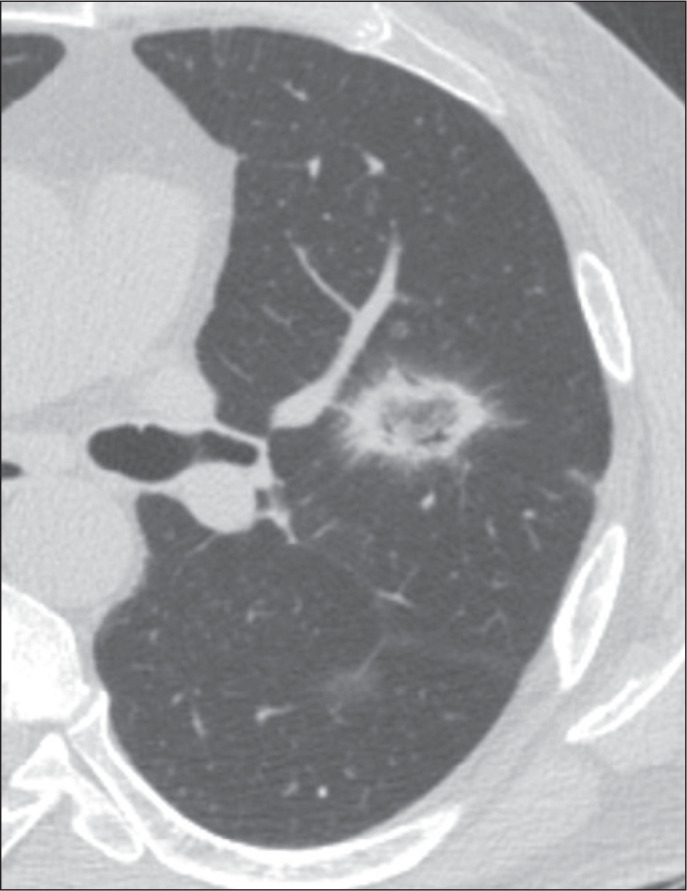
Example of a smooth-walled, centrally located RHS in a patient with cryptogenic organizing pneumonia.

**Figure 2:**
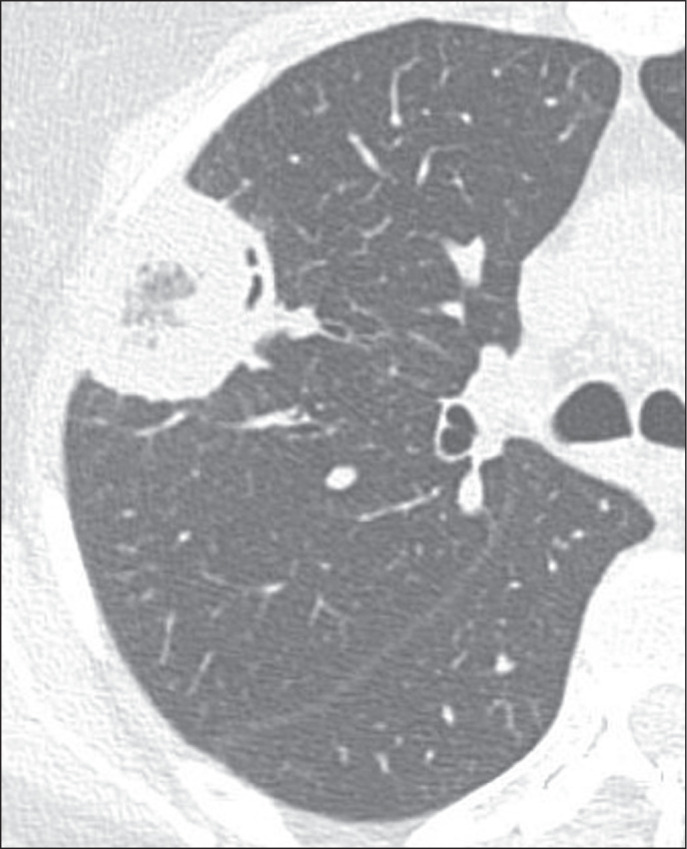
Example of an RHS with a thick (> 1 cm) wall and a peripheral location in a patient with fungal infection (mucormycosis).

**Figure 3:**
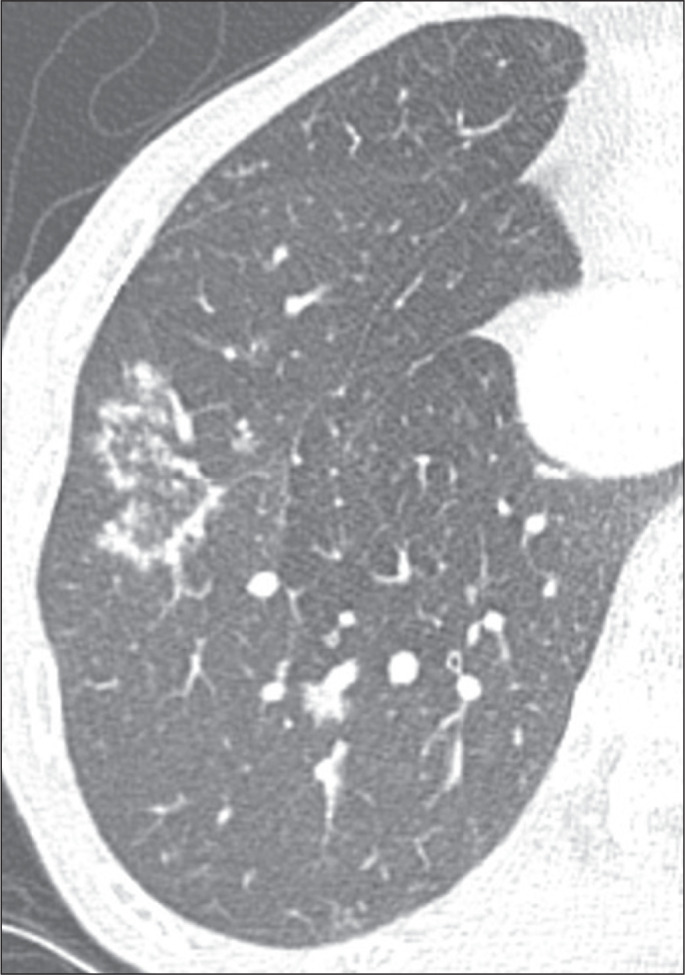
Example of an RHS with a nodular wall and a peripheral location in a patient with fungal infection (paracoccidioidomycosis).

The criteria for characterizing the CT findings were defined according to the Fleischner Society Glossary of Terms^(8)^. Cases containing more than one reversed halo were characterized as cases of multiple RHSs. If the RHS was located within 2 cm of any pleural surface, it was defined as a peripheral RHS. In cases with both distributions (central and peripheral), the predominant location was considered. Regarding the thickness of the RHS, consolidation halos with a thickness ≥ 1 cm were defined as thick RHSs^(4)^. Consolidation halos with rounded or nodular areas on their walls were characterized as nodular RHSs, unlike the smooth RHSs, which did not present that characteristic (Figures 1, 2, and 3). Cases with possible discrepancies in the descriptors were resolved by a third thoracic radiologist, with 10 years of experience. Cases with no reversed halo on the images were excluded, as were those for which there were no images in the system and those in which there was no definitive diagnosis.

This study was approved by the local research ethics committee (Reference no. 7,334,669). Because the data were collected retrospectively, the requirement for informed consent was waived.

## RESULTS

We identified 343 chest CT reports containing the term “reversed halo”. Of those, 57 were excluded, for the following reasons: absence of an RHS in the images (n = 6); absence of images of the examination in the system (n = 15); and lack of a definitive final diagnosis (n = 36). Therefore, the final sample comprised 286 examinations.

**Figure 4:**
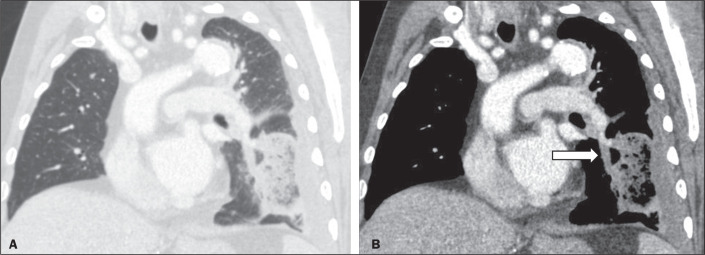
Oblique CT images showing an RHS in a lung window (A) and pulmonary thromboembolism in a mediastinal window (B). In this case, the RHS was in an area of pulmonary infarction.

**Figure 5:**
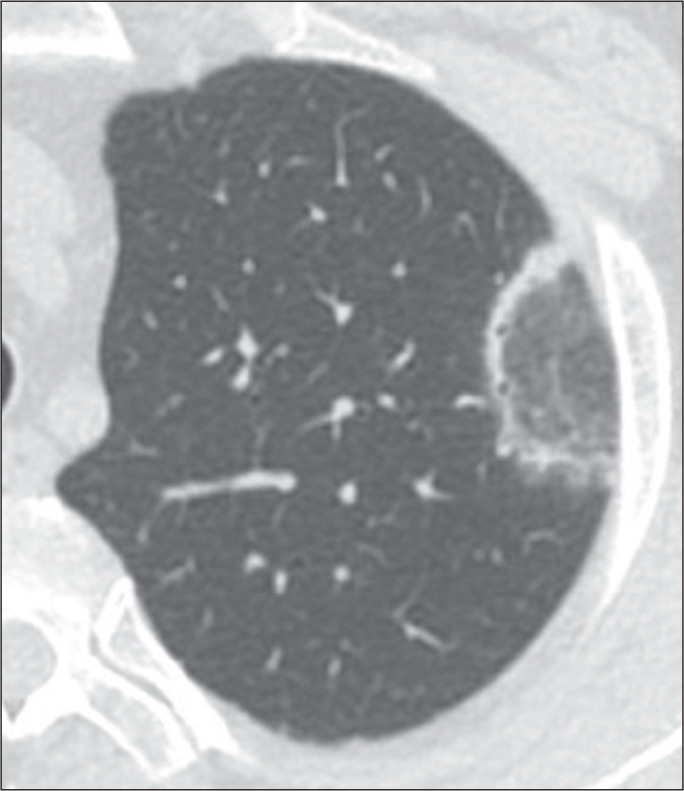
Another example of a smooth-walled RHS in a patient with cryptogenic organizing pneumonia.

**Figure 6:**
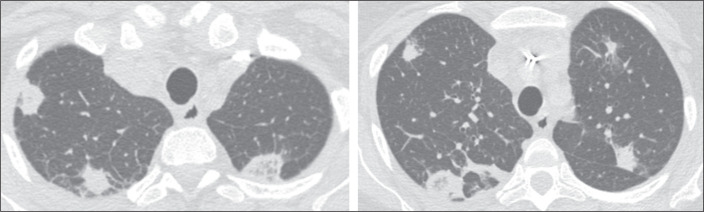
Multiple peripherally located RHSs in a patient with septic embolism resulting from central venous catheter-related infection.

**Figure 7:**
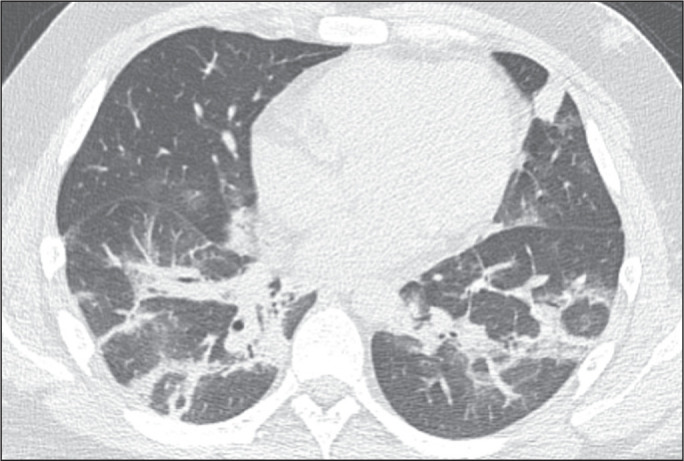
Patient with COVID-19 presenting with bilateral ground-glass opacities, some with an RHS conformation.

**Figure 8:**
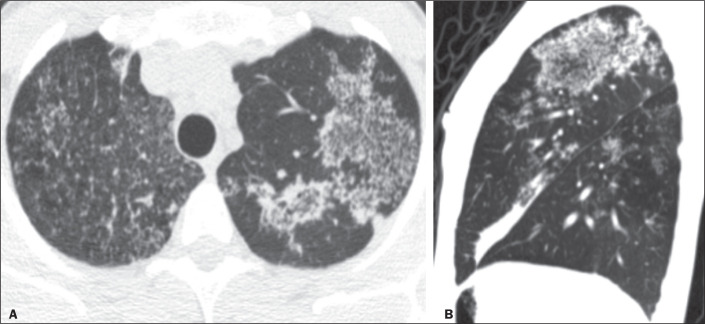
CT images in the axial and sagittal planes (A and B, respectively), showing multiple pulmonary micronodules, sometimes forming an RHS with nodular borders, in a patient with tuberculosis.

**Figure 9:**
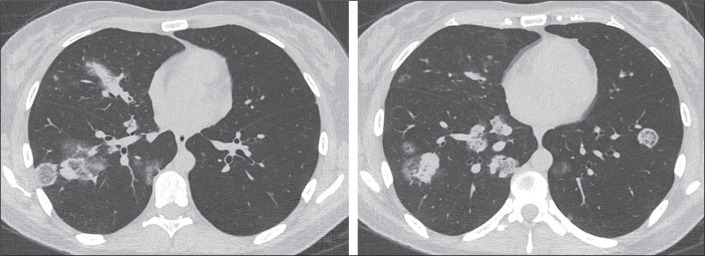
Patient with adenoid cystic carcinoma of the trachea and pulmonary metastases, presenting with an RHS.

Among the 286 examinations, the final diagnosis was pulmonary infarction in 120 (42.0%), organizing pneumonia in 49 (17.1%), bacterial pneumonia in 46 (16.1%), viral pneumonia in 40 (14.0%), granulomatous infections (tuberculosis and fungal infections) in 17 (5.9%), and other causes, designated the miscellaneous group (which included cases of lung metastases, squamous cell carcinoma, lymphangitic carcinomatosis, and pneumonitis due to treatment of lung neoplasia) in 14 (4.9%). In the viral pneumonia group, there were 30 cases of COVID-19, representing 10.5% of the sample. The results are summarized in Table 1, and the illustrative images are shown in Figures 4 to 9.

**Table 1:** RHS etiologies in the study sample (N = 286).

Diagnosis	CT finding of an RHS n (%)
Pulmonary thromboembolism	120 (41.9)
Cryptogenic organizing pneumonia	49 (17.1)
Bacterial pneumonia	45 (15.7)
Viral pneumonia	39 (13.6)
Granulomatous diseases	18 (6.2)
Miscellaneous	15 (5.2)

In our sample, pulmonary infarction secondary to pulmonary thromboembolism was the most prevalent disease with an RHS, seen in 120 cases (42.0%), with the most common specific characteristics being the presence of a smooth halo (in 81.6%), an almost exclusively peripheral distribution (in 99.1%), and a solitary character (in 65.8%). Bacterial and viral infections together represented the second most prevalent cause of an RHS, accounting for nearly 30% of cases. The findings were nonspecific, and the prevalence of a solitary RHS was 57.6% in bacterial etiologies, higher than in other infectious etiologies. Some bacterial etiologies included isolation of strains of the genera *Streptococcus, Staphylococcus, Klebsiella*, and *Pseudomonas*. Cryptogenic organizing pneumonia was identified in 49 (17.1%) of the 286 cases, with the main characteristics of the RHSs being the presence of a smooth halo (in 81.6%), a mostly peripheral distribution (in 91.8%), and multiple locations (67.3% of cases). Granulomatous diseases were an uncommon cause of an RHS, seen in only 18 (6.3%) of cases, mostly represented by fungal infections, which accounted for 12 of those cases, with the remainder all being cases of infection with *Mycobacterium tuberculosis*. A nodular halo was observed in nine (50.0%) of those 18 cases, 13 (72.2%) of which had a peripheral distribution. The miscellaneous group totaled 15 cases, the great majority of which (73.3%) were cases of pulmonary metastases, followed by radiofrequency ablation of metastases (6.7%), squamous cell carcinoma (6.7%), lymphangitic carcinomatosis (6.7%), and pneumonitis due to treatment of primary lung neoplasia (6.7%). The specific characteristics are presented in Tables 2 and 3.

**Table 2:** Characteristics of the RHS walls among different etiologies in the study sample (N = 286).

Diagnosis	Wall characteristic		
	Smooth n (%)	Thick n (%)	Nodular n (%)
Pulmonary thromboembolism	98 (81.6)	20 (16.6)	2 (1.8)
Cryptogenic organizing pneumonia	40 (81.0)	5 (10.2)	4 (8.8)
Bacterial pneumonia	29 (64.5)	13 (28.9)	3 (6.6)
Viral pneumonia	29 (74.3)	8 (20.5)	2 (5.2)
Granulomatous diseases	7 (38.9)	2 (11.1)	9 (50.0)
Miscellaneous	4 (26.5)	5 (33.3)	6 (40.8)

**Table 3:** Distribution and number of RHSs among different etiologies in the study sample (N = 286).

Diagnosis	Distribution		Number	
	Peripheral n (%)	Central n (%)	Solitary n (%)	Multiple n (%)
Pulmonary thromboembolism	119 (99.1)	1 (0.9)	79 (65.8)	41 (34.2%)
Cryptogenic organizing pneumonia	45 (92.0)	4 (8.0)	16 (32.6)	33 (67.4%)
Bacterial pneumonia	41 (91.1)	4 (9.9)	26 (57.7)	19 (42.3%)
Viral pneumonia	36 (92.0)	3 (8.0)	4 (10.0)	35 (90.0%)
Granulomatous diseases	13 (72.3)	5 (27.7)	9 (50.0)	9 (50.0%)
Miscellaneous	13 (87.0)	2 (13.0)	11 (73.3)	4 (26.7%)

## DISCUSSION

The RHS was first described in 1996 by Voloudaki et al.^(2)^ in an article that characterized the chest CT findings of two cases of cryptogenic organizing pneumonia. In that article, the currently accepted term was not used, and the finding was described as a crescent-shaped or ring-shaped opacity with adjacent ground-glass areas. The authors reported that in the histopathological analysis, the central ground-glass opacities corresponded to areas of inflammation near the alveolar septa and to cellular debris, whereas the peripheral consolidation corresponded to areas of organizing pneumonia in the alveolar ducts. In 1999, the sign was described by Zompatori et al.^(9)^ in a case report of cryptogenic organizing pneumonia, in which it was characterized as the atoll sign, in reference to the similarity of the finding with atolls, which consist of ring-shaped oceanic islands with a lagoon inside. In that same case report, the authors noted that it resembled the halo sign but was inverted. The currently accepted term came into use in 2003, after Kim et al.^(10)^ defined it in a study that evaluated the presence of this finding in patients with cryptogenic organizing pneumonia. Currently, according to the Fleischner Society glossary of thoracic imaging terms^(8)^ and the consensus on thoracic radiology terminology in the Portuguese of Brazil and Portugal^(1)^, the sign is defined as the presence of focal ground-glass opacity surrounded by a complete or partial ring of consolidations.

In 2005, Gasparetto et al.^(11)^ described the finding in patients with paracoccidioidomycosis, indicating that the RHS is not exclusive to organizing pneumonia. Since then, various authors have described the RHS in other infectious and noninfectious conditions. Since it has been associated with different etiologies, some studies have evaluated characteristics that could aid in the differential diagnosis of this sign, considering morphological characteristics (such as a halo with smooth, thick, or micronodular edges) and in relation to the distribution of the finding (whether solitary or multiple, central or peripheral).

In the present study, noninfectious etiologies were the most common causes of RHSs, with pulmonary infarction secondary to pulmonary thromboembolism being the main cause, accounting for 42% of the cases evaluated. In most cases of pulmonary thromboembolism, the RHS was solitary, with a peripheral location and a smooth halo. Other studies have also evaluated the presence of an RHS related to pulmonary thromboembolism. A study conducted by Marchiori et al.^(12)^ in 2017 identified at least one RHS in 64 (15.9%) of 402 patients testing positive for acute pulmonary thromboembolism. Those authors also found that most (84.35%) of the RHSs were solitary, as well as that 93.24% were located in the lower lung fields and that 95.95% were located in the periphery of the lung^(12)^. These findings suggestive of pulmonary infarction are of great relevance because even examinations without contrast can raise the suspicion of pulmonary thromboembolism, a clinically urgent condition with high morbidity and mortality.

In the present study, cryptogenic organizing pneumonia was the second leading noninfectious cause of RHS. This result differs from those of other studies that evaluated the causes of RHS. In 2012, Marchiori et al.^(13)^ evaluated 79 consecutive cases presenting with RHS on chest CT, dividing the cases between infectious and noninfectious causes. In that study, the most common noninfectious cause of RHS was organizing pneumonia, followed by pulmonary thromboembolism, the latter occurring in 7 of 38 patients^(13)^. In another retrospective study, conducted in 2015, Zhan et al.^(14)^ evaluated 108 cases in which chest CT showed RHS, as well as their respective causes. In that study, organizing pneumonia was also identified as the main cause not related to granulomatous diseases, with an RHS being identified in 24% of cases. However, those authors did not comment on the prevalence of the finding related to acute pulmonary thromboembolism.

For immunocompromised patients who present with an RHS on CT, infectious causes are among the most important differential diagnoses, with tuberculosis and fungal infections being the most common causes^(13)^. In a systematic review conducted in 2014, Maturu et al.^(6)^ suggested that the presence of an RHS in immunocompromised patients should raise suspicion of fungal infections, including invasive ones, especially mucormycosis. In that systematic review, the RHSs related to invasive fungal infections in immunocompromised patients were characterized as one or more large lesions, a pattern that was also seen in the case of mucormycosis seen in our study. The RHS has also been described in endemic infections, such as tuberculosis and paracoccidioidomycosis, and in these cases it is characterized by bilateral, asymmetric lesions, together with centrilobular nodules, consolidations, and ground-glass opacities.

In the present study, half of the RHSs in patients with granulomatous diseases had a nodular halo morphology and most of those patients had fungal infections, the remainder having tuberculosis. In those cases, the RHSs also more commonly had a peripheral location. Other studies have also associated granulomatous diseases with the nodular halo morphology of an RHS. A review of the literature conducted by Marchiori et al.^(15)^ related this characteristic to the presence of granulomas, a finding corroborated by the pathology analysis described by Zhan et al.^(14)^. In addition to granulomatous diseases, the nodular morphology of the RHS was also identified in our group of miscellaneous causes (mainly metastases), with a prevalence of 40% (in a total of 15 cases) in that group. Sarcomas and squamous cell carcinomas were the primary tumors that presented this pattern of metastasis in our study.

Our study has some limitations. It was a single-center study conducted at a tertiary care hospital that is a referral center for highly complex cases. Therefore, the results obtained might not be generalizable to the general population, which could explain aspects such as the high number of cases with a final diagnosis of pulmonary thromboembolism. In all cases, the final diagnosis was made by reviewing the electronic medical records, evaluating the laboratory test results, and analyzing the pathology findings when available, as well as by reviewing the subsequent follow-up imaging examinations. Patients without a definitive diagnosis were excluded. Although it was a retrospective study, the inclusion of consecutive cases over a five-year period reduced the possibility of selection bias. Despite these considerations, our study represents one of the largest case series in the literature on the RHS, evaluating cases with different etiologies, as well as the CT characteristics of the sign.

## CONCLUSION

Although initially described in cases of organizing pneumonia, the RHS is a nonspecific finding that can occur in various diseases. Nevertheless, some characteristics of its presentation can help narrow the differential diagnosis, especially when correlated with the clinical context. For example, the presence of a solitary peripheral RHS is suggestive of acute pulmonary thromboembolism with pulmonary infarction and, if the chest CT was performed without contrast, a complementary evaluation with computed tomography angiography of the pulmonary arteries is recommended. However, the presence of an RHS in an immunocompromised patient should raise suspicion of infectious causes, including fungal infection and tuberculosis. When an RHS with micronodular morphology is identified, granulomatous etiologies should be suspected, and other causes, such as metastases, should also be considered.

Although an RHS is often nonspecific, some CT characteristics, when taken together with clinical and laboratory data, can help determine the differential diagnosis. Familiarity with the identification of this sign, its CT characteristics, and its differential diagnoses is of great importance to radiologists, promoting better case management.
